# Improving the performance of single-cell RNA-seq data mining based on relative expression orderings

**DOI:** 10.1093/bib/bbac556

**Published:** 2022-12-18

**Authors:** Yuanyuan Chen, Hao Zhang, Xiao Sun

**Affiliations:** State Key Laboratory of Bioelectronics, School of Biological Science and Medical Engineering, Southeast University, Nanjing 210096, China; College of Science, Nanjing Agricultural University, Nanjing 210095, China; College of Science, Nanjing Agricultural University, Nanjing 210095, China; State Key Laboratory of Bioelectronics, School of Biological Science and Medical Engineering, Southeast University, Nanjing 210096, China

**Keywords:** single-cell analysis, relative expression orderings, interaction/edge-level features, delta rank matrix, cell-specific network

## Abstract

The advent of single-cell RNA-sequencing (scRNA-seq) provides an unprecedented opportunity to explore gene expression profiles at the single-cell level. However, gene expression values vary over time and under different conditions even within the same cell. There is an urgent need for more stable and reliable feature variables at the single-cell level to depict cell heterogeneity. Thus, we construct a new feature matrix called the delta rank matrix (DRM) from scRNA-seq data by integrating an a priori gene interaction network, which transforms the unreliable gene expression value into a stable gene interaction/edge value on a single-cell basis. This is the first time that a gene-level feature has been transformed into an interaction/edge-level for scRNA-seq data analysis based on relative expression orderings. Experiments on various scRNA-seq datasets have demonstrated that DRM performs better than the original gene expression matrix in cell clustering, cell identification and pseudo-trajectory reconstruction. More importantly, the DRM really achieves the fusion of gene expressions and gene interactions and provides a method of measuring gene interactions at the single-cell level. Thus, the DRM can be used to find changes in gene interactions among different cell types, which may open up a new way to analyze scRNA-seq data from an interaction perspective. In addition, DRM provides a new method to construct a cell-specific network for each single cell instead of a group of cells as in traditional network construction methods. DRM’s exceptional performance is due to its extraction of rich gene-association information on biological systems and stable characterization of cells.

## Introduction

The types, states and interactions between human tissues cells vary enormously [1]. Single-cell RNA-sequencing (scRNA-seq) provides a method to examine gene expression at the single-cell level to better study these tissues, the different cell types present in them, and the heterogeneity and functional diversity among them [2–5]. A growing number of studies have designed many effective computational methods to cluster cells, identify new cell types and construct pseudo-trajectory by solving the problems of high noise and sparsity, low coverage, the batch effect and dropout events in scRNA-seq [6–8].

However, most current single-cell analysis methods mainly focus on gene expression values, which may be different if measured at different time points or under different conditions, even in the same cell. Gene–gene interactions and networks, on the other hand, are more stable and reliable and can regulate gene expression [9, 10]. Furthermore, the addition of network information reduces the impact of expression noise, which has motivated many scholars to consider network fusion in single-cell analysis. Gene interactions information and priori networks and pathways have been integrated into some computational methods to improve accuracy and robustness [11–13], and many integrated network methods have shown that gene interactions or networks are beneficial to cell-type identification and other analyses. A pathway and gene set overdispersion analysis method, PAGODA, was developed to resolve multiple, potentially overlapping aspects of transcriptional heterogeneity by testing gene sets for coordinated variability among measured cells [14]. Wang *et al*. [12] showed that pathway signals extracted from scRNA-seq data help classify and cluster heterogeneous cells. Zhang *et al*. [15] showed that integrating pathways can significantly improve the accuracy and robustness of most single-cell clustering methods with an effective integration framework.

Although these methods perform well in single-cell analysis, the real interaction between genes is ignored. Most methods use the gene set in networks or pathways to construct networks limited to the grouped cells instead of each individual cell. While scRNA-seq may give more information of an insight into the gene–gene interaction networks based on the sequencing of a large number of cells. This motivated us to consider the possibility of using edge-level features (i.e. gene-interaction features) for each cell to improve the existing single-cell analysis method, and to further consider the construction of cell-specific networks to characterize cell heterogeneity from a gene pair perspective.

In this study, we propose a new feature matrix delta rank matrix (DRM) for single-cell analysis basis on scRNA-seq using relative expression orderings. Once a gene expression matrix (GEM) is obtained, by integrating an a priori gene interaction network, we transform it into a DRM matrix containing gene–gene association information. The rows and columns of the DRM are the edges (or connected gene pairs) in the network and cells, respectively. By DRM, it is the first time that we transform the gene-level feature into edge-level for scRNA-seq data, thereby turning the ‘unreliable’ information into ‘reliable’. All additional analyses including dimension-reduction, cell clustering, cell-marker identification and pseudo-trajectory reconstruction on the GEM can also be performed on the DRM from an edge perspective by using any existing scRNA-seq analysis method.

Experiments on various scRNA-seq datasets have shown DRM to be more accurate and robust than the original GEM using most clustering methods evaluated by adjusted rand index (ARI), normalized mutual information (NMI) and purity. The DRM is better than the GEM at distinguishing similar types of cells. In addition, DRM can find changes in gene interactions among different cell types. DRM also provides a new method to construct a cell-specific network for each single cell using only the expression profile of the cell without requiring reference cells.

In summary, our constructed feature matrix DRM provides a new strategy to analyze the scRNA-seq data and really achieves the fusion of gene expressions and gene interactions. Users of this method can achieve better results by first transforming the GEM into DRM, and then performing downstream analysis on DRM. The DRM can extract richer information about biological systems at the gene interaction level, which may open new avenues for the analysis of scRNA-seq data from an interaction perspective.

## Materials and methods

### Stability of the relative expression ordering

In this paper, we introduce a new feature for single-cell analysis called the DRM, which we derived from the single-cell GEM and an a priori background network based on relative expression orderings.

To determine whether the relative expression ordering of gene pairs is stable for a particular type of cells, scRNA-seq data of normal human liver tissues from four different datasets using different sequencing platforms were collected [16–19]. We randomly selected 50 hepatocyte cells from each of the GSE124395, GSE115469 and GSE149614 databases, and 36 hepatocyte cells from GSE136103. Brief introductions to and sources of all datasets are listed in Supplementary Table S1.

**Figure 1:**
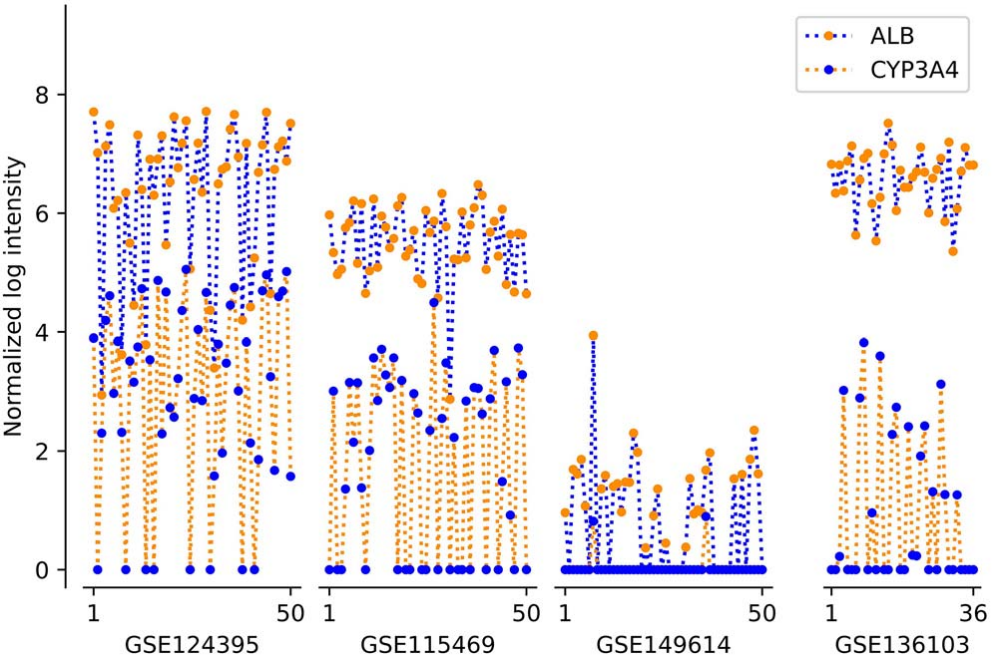
An example of gene pair (*ALB* and *CYP3A4*) showing stable relative expression orderings in normal human hepatocyte cells. The plot illustrates the expression distributions of genes *ALB* and *CYP3A4* in hepatocyte cells from different datasets are still different even after LogNormalization. For example, the expression levels of these two genes in all hepatocyte cells of GSE124395 are always higher than those in GSE149614. However, the relative expression ordering of gene *ALB* is always larger than that of gene *CYP3A4* within each cell in any of the datasets.

We selected pair of genes (*ALB* and *CYP3A4*) with stable expression ordering obtained from hepatocyte cells as an example to show the inherent advantage of stable relative expression ordering of gene pairs over distribution variations of gene expression. Even after the same normalization (LogNormalize), the expression distributions of the two genes in hepatocyte cells from different datasets remain different (Figure 1). Thus, it is not possible to compare expression levels of genes in different datasets, and data integration from different databases is difficult. However, the relative ordering of gene expression within each cell is stable. The expression level of gene *ALB* stays at least as high as gene *CYP3A4* within each cell across all the datasets. Thus, relative expression ranking (i.e. the difference between gene expression ranking) can be used as a more stable feature to characterize a single cell. These results provide a basis for constructing reliable cell-specific features for single-cell analysis using relative expression ranking information.

### Construction of the DRM

**Figure 2:**
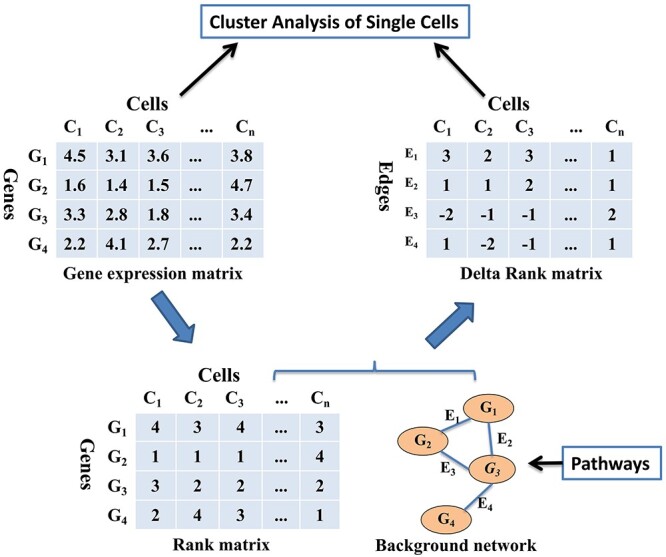
Schematic illustration of DRM construction. (i) Convert the GEM with *m* genes and *n* cells into a rank matrix by ranking all genes according to gene expression values. Here, we take *m = 4* as an example. (ii) Collect gene–gene interaction networks, such as gene association networks in pathways, as a priori background network, which consists of *l* edges. Here, we take *l = 4* as an example. (iii) Integrate the rank matrix and the background network to construct the DRM, which is composed of *l* rows and *n* columns. Its rows and columns correspond to edges and cells, respectively, and it can be analyzed using any existing scRNA-seq analysis method.

Given a single-cell RNA-seq dataset with $m$ genes and $n$ cells and a background network with $l$ edges, we can construct a DRM with $l$ rows and $n$ columns (Figure 2). This process converts the gene-level information into edge-level information at the single-cell level, allowing the characterization of a single cell in a biological system.

The DRM construction process has three steps (Figure 2): (i) construction of the rank matrix; (ii) collection of an a priori background network and (iii) construction of the DRM. First, each gene expression value is converted into its rank within each cell (the smallest expression value corresponds to the lowest rank, and the largest expression value corresponds to the highest rank). As a result, the expression matrix is converted into a rank matrix (denoted by R with element ${r}_{i,c}$, which represents the rank of gene ${g}_i$ in cell $c$ by ranking all genes according to the expression values in all cells. After collecting the background network, which consists of $l$ edges (i.e. interaction gene pairs), the DRM can be calculated by subtracting the ranks of each pair of genes connected by an edge as follows: for a given cell $c$

$$ {\varDelta}_{e,c}={r}_{i,c}-{r}_{j,c}, $$

where genes ${g}_i$ and ${g}_j$ are connected by edge $e$ in the background network. Finally, z-score normalization is performed for each column of DRM

$$ \mathrm{Z}-\mathrm{score}=\frac{X-\overline{X}}{\sigma } $$

and the normalized matrix DRM if dimension$l\ast n$is obtained. The rows and columns of DRM represent edges in the background network and cells, respectively. All analyses in the current study on the DRM are on the normalized DRM unless noted otherwise.

### Background network used in DRM construction

The a priori background network used in the construction of the DRM was downloaded from the R package ‘ESEA’ [20]. The ‘ESEA’ package constructs a background set of edges by extracting human pathway structures (e.g. interaction, regulation, modification and binding) from seven public databases (including KEGG, Reactome, Biocarta, NCI, SPIKE, HumanCyc and Panther) and the edge sets of pathways for each of the above databases. In total, 2328 pathways containing 164 862 edges (i.e. interaction gene pairs) without duplication were extracted. All 164 862 edges were integrated into a final large network as the background network.

### Datasets used for validation of the DRM

In this work, we collected eight high-quality datasets from the literatures (including Chu-type dataset [21], Chu-time dataset [21], Yan dataset [22], Yan2 dataset [22], Camp dataset [23], Pollen dataset [24], Pollen dataset [25] and Calzetti dataset [26]) to validate the advantages of our new feature matrix DRM. These datasets include human embryonic stem cells, cortical cells, bone marrows, etc. To ensure the fairness of comparison, cell types in most datasets are clearly defined by the different cell sources (e.g. blood cells, neural cells) or different time points. For other datasets, FACS (fluorescence-activated cell sorting) assays were used to identify cell types, which also ensure high quality of data. Brief introductions and sources of all datasets are listed in Supplementary Table S2 and Supplementary Note S1.

### Clustering and pseudo-trajectory analysis

Since we construct our new feature matrix, the DRM, from the GEM, all the further analyses including dimension-reduction, clustering, cell-marker identification and pseudo-trajectory construction normally performed on the GEM can be performed on DRM from an edge (gene interaction) perspective using any existing scRNA-seq analysis method. We selected several scRNA-seq analysis methods to compare the performance of the DRM and GEM. Notably, we focused on the comparison of the traditional feature matrix (GEM) to our new feature matrix (DRM), but not on the clustering methods themselves. Accordingly, we used the same set of values for all the parameters in the methods for the GEM and DRM unless noted otherwise. We generally used the default parameters, which are listed in Supplementary Note S2.

We used seven classical clustering methods (including SC3 [27], Seurat [3], spectral [28], hierarchical [29], DBSCAN [30], kmeans [31] and pcaReduce [32]) to perform cell clustering analysis. The details of the cell clustering methods used in this paper are introduced in Supplementary Note S3.

Since the classification of each cell was known, we used the ARI, NMI and purity to evaluate the accuracy of clustering methods comparing DRM and GEM. ARI measures the degree of coincidence between two distributions and ranges from −1 to 1. The closer the value is to 1, the more consistent the clustering result is with the true state. NMI measures the similarity of two clustering results and ranges from 0 to 1. Higher NMI values indicate higher similarity between the two clustering results. Purity is defined as the proportion of the correct clustering number to the total number and ranges from 0 to 1. Higher purity values indicate higher clustering accuracy.

We used Monocle3 [33] in the pseudo-trajectory analysis, which first projects cells onto a low-dimensional space encoding transcriptional state using UMAP [34]. It resolves the paths or trajectories that individual cells can take during development and gives each cell a pseudotime value to represent the cells’ differentiate order; cells in the later stages are assigned larger values. The pseudo-trajectory analysis was performed through four main steps: normalization, dimension reduction, single-cell clustering and pseudotime trajectory diagram learning. The parameters in Monocle3 were set to default.

### Dimension-reduction and feature-edge selection

The top $k$ ($k$ = 50, 100, 150, 200, 250, 300, 350, 400, 450, 500, 600, 700, 800, 900) genes (or edges) with the highest variance based on the GEM (or DRM) were selected for cluster analysis. The optimal $k$ can be determined by using the above clustering method and evaluation indexes.

### Cell marker identification

Cell-type specific gene signatures (i.e. cell marker genes) can be identified by significant difference analysis. We used a similar strategy to identify cell-type specific edge signatures called cell marker edges. Edges significantly differing between a population and all other populations in terms of delta rank values were identified using a two-sided unequal variance t-test. Edges with an false discovery rate (FDR) < 0.01 were considered significant. For each cell type, significant edges were ordered by decreasing fold change FD

$$ {FD}_{e,t}=\left|{\overline{\varDelta}}_{e,c=t}-{\overline{\varDelta}}_{e,c\ne t}\right| $$

compared with other cell subsets, where ${\overline{\varDelta}}_{e,c=t}$ is the mean delta rank among cell type $t$ for edge $e$ and ${\overline{\varDelta}}_{e,c\ne t}$ is the mean delta rank of all cells other than type $t$. The top $k$ significant edges from each cell type were combined into cell marker edges. We chose $k$ such that the number of cell marker edges is close to the number of cell marker genes for comparability.

### Construction of a cell-specific network

In this section, we propose a new method to construct a cell-specific network based on the delta rank of the edges of each cell. A Monte Carlo test was used to estimate the empirical *P*-value corresponding to each delta rank value ${\Delta}_{\mathrm{e},\mathrm{c}}$. Specifically speaking, we derived the statistical significance of the delta rank value in the DRM based on an empirical NULL distribution (Supplementary Figure S1) generated by 10 000 random shuffles. Edges with a *P*-value <0.05 are considered to be significant. All significant edges for a cell constitute a cell-specific network. For a fixed cell type, we integrated the cell-specific networks into a cell-type-specific network by calculating the mean value of the delta rank of the edges.

## Results

### Cell clustering and feature dimension-reduction analysis on DRM

**Figure 3:**
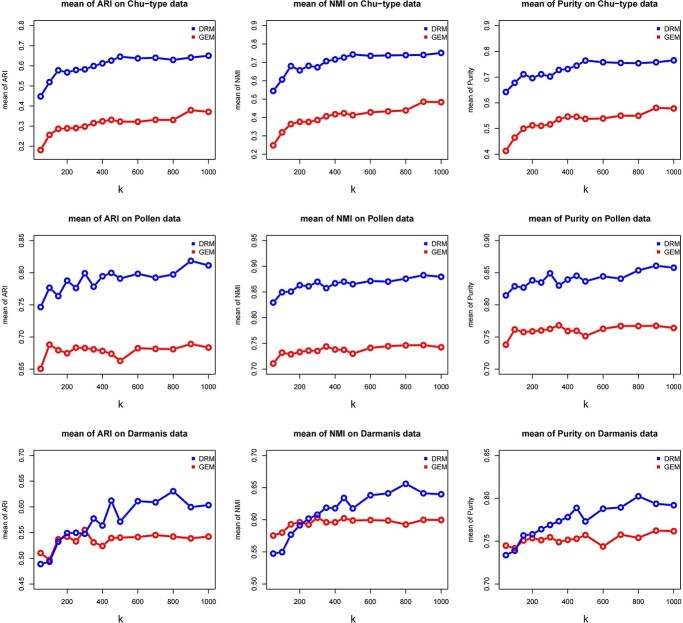
The clustering performances of the DRM and GEM based on differing numbers of features in the Chu-type, Pollen and Darmanis datasets. The blue and red points represent the performance of DRM and GEM features, respectively. The top *k* (*k* = 50, 100, 150, 200, 250, 300, 350, 400, 450, 500, 600, 700, 800, 900) genes (or edges) with the largest variance were selected for cluster analysis. The average performance of the above seven clustering methods (introduced in the Materials and Method section) on the three databases is shown on the vertical axis.

Different numbers of feature genes (or edges) with large variances were selected for cluster analysis. The average performance on ARI, NMI and purity of the above seven algorithms (for details, see the Materials and Methods section) on the Chu-type, Pollen and Darmanis datasets is shown in Figure 3. The clustering results were significantly improved with an increase in the number of feature genes or feature edges. The ARI, NMI and purity indices of clustering converged when the number of features, $\mathrm{k}$, increased to 500. Therefore, we selected the top 500 features for subsequent cluster comparison. The performance based on the DRM cluster was significantly better than on the GEM according to three different evaluation indices for different numbers of clustering features.

**Figure 4:**
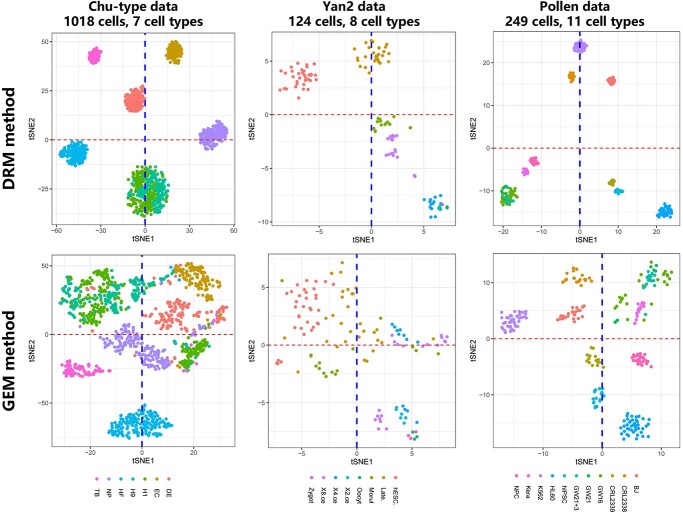
The clustering performances of the DRM and GEM on the Chu-type, Yan and Pollen datasets. t-SNE plots are used for visualization and different colors represent different cell types.

**Figure 5:**
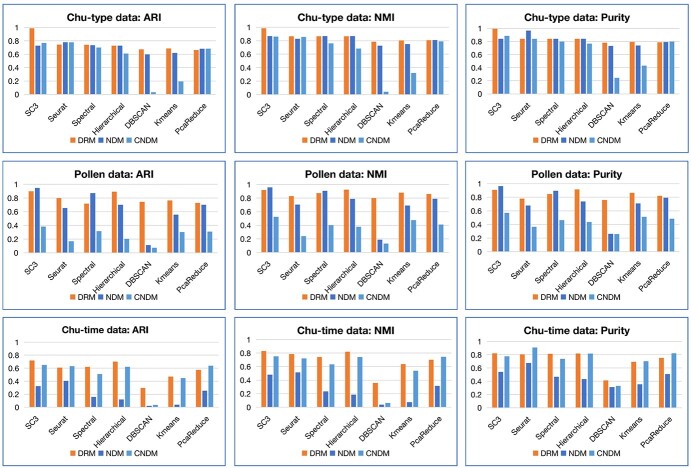
The comparison of the DRM, NDM and CNDM in cell clustering analysis (using the top 500 features), evaluated by ARI, NMI and Purity on the Chu-type data, Pollen data and Chu-time datasets.

**Figure 6:**
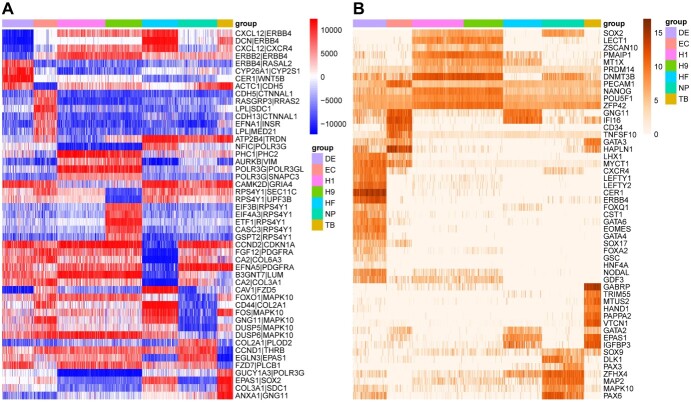
(**A**) Heatmap of edge delta rank for 49 cell marker edges for 1018 cells of 7 cell types. High to low delta rank value corresponds to green to red in the heatmap. (**B**) Heatmap of expression of gene markers. High to low expression corresponds to dark orange to light orange in the heatmap. Seven cell types are color-coded by the band on the top of the heatmap.

**Figure 7:**
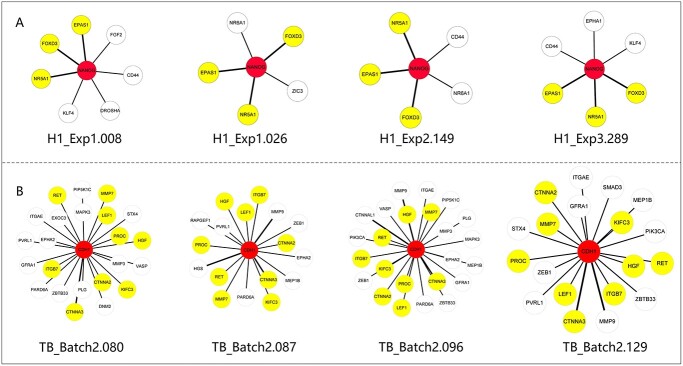
Cell-specific networks characterize cell-specific features and reveal common network patterns for cells of the same type. (**A**) The four cell-specific subnetworks of the *NANOG* gene from four H1 cells. The numbers of the connections with *NANOG* for the four cells are respectively 7, 5, 5 and 6, and the genes linked to *NANOG* are different among the four H1 cells. However, E *PAS1*, *FOXD3* and *NR5A1* (the yellow color) are common genes appearing in the four subnetworks. (**B**) The four cell-specific subnetworks of the *CDH1* gene from four TB cells. The numbers of the connections with *CDH1* for the four cells are 23, 17, 24 and 19, and the genes linked to *CDH1* are different in the four TB cells. However, *RET*, *LEF1*, *MMP7*, *PROC*, *HGF*, *CTNNA3*, *CTNNA2*, *KIFC3* and *ITGB7* (the yellow color) are common genes appearing in the four subnetworks.

**Figure 8:**
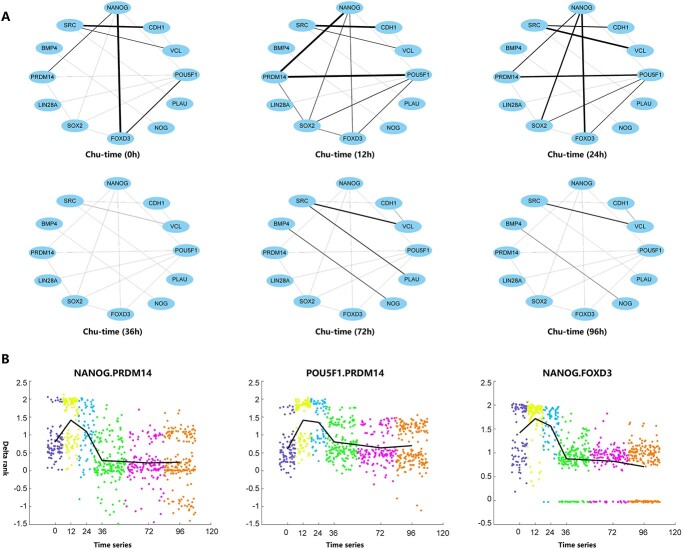
(**A**) Subnetworks of some single cells with the 12 genes that are involved in human embryo development, where the larger mean value of normalized deltarank leads to a darker edge. (**B**) The delta rank of *NANOG*&*PRDM14*, *POU5F1*&*PRDM14* and *NANOG*&*FOXD3* along the six time points of embryo development in the Chu-time dataset. The solid black lines represent the lines formed by the average value of the delta rank of all cells at the six time points of embryo development.

**Figure 9:**
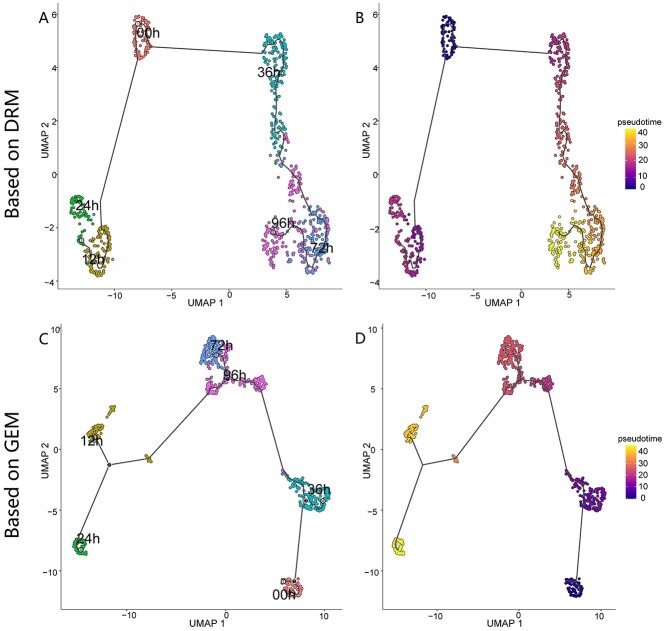
(**A**) Pseudotime trajectory of human embryonic stem cells based on DRM (cell number, *n* = 758), colored by the gold standard development stages. (**B**) Pseudotime trajectory of human embryonic stem cells based on the DRM, colored by the predicted pseudotime derived from the DRM. (**C**) Pseudotime trajectory of human embryonic stem cells based on the GEM, colored by the gold standard development stages. (**D**) Pseudotime trajectory of human embryonic stem cells based on the GEM, colored by the predicted pseudotime derived from GEM. The brighter points represent the end of the cell differentiation in (B) and (D).

Based on the new feature matrix DRM, we performed clustering analysis using the seven clustering algorithms and t-distributed stochastic neighbor embedding (t-SNE) [35] which represents a nonlinear method for performing dimension-reduction analysis. The GEM and DRM were used for comparison on all eight datasets (Supplementary Table S2) using the same algorithm parameters. The performance of the new DRM was superior to the original GEM for various methods on various datasets as evaluated by ARI, NMI and purity (Table 1 and Supplementary Tables S3 and S4).

**Table 1:** The comparison of GEM and DRM in clustering analysis, evaluated by ARI

		**Chu-type**	**Chu-time**	**Yan**	**Yan2**	***Camp***	***Pollen***	***Darmanis***	***Calzetti***
**SC3**	**GEM**	0.78	**0.73**	0.66	0.61	**0.76**	**0.93**	**0.90**	0.27
	**DRM**	**0.99**	0.71	**0.80**	**0.76**	0.74	0.90	0.79	**0.40**
**Seurat**	**GEM**	0.66	0.55	0.69	0.70	0.72	0.79	0.75	0.11
	**DRM**	**0.77**	**0.61**	**0.69**	**0.78**	**0.73**	**0.80**	**0.86**	**0.11**
**Spectral**	**GEM**	0.30	0.21	**0.93**	0.81	**0.68**	0.62	0.43	0.27
	**DRM**	**0.75**	**0.48**	0.88	**0.94**	0.66	**0.72**	**0.53**	**0.43**
**Hierarchical**	**GEM**	0.21	0.20	**0.89**	0.68	0.74	0.89	0.57	0.24
	**DRM**	**0.76**	**0.69**	0.64	**0.78**	**0.74**	**0.89**	**0.64**	**0.51**
**DBSCAN**	**GEM**	0.08	0.01	0.58	0.18	0.18	0.06	**0.08**	**0.07**
	**DRM**	**0.66**	**0.31**	**0.66**	**0.72**	**0.19**	**0.74**	0.03	0
**kmeans**	**GEM**	0.15	0.15	0.67	0.52	0.55	0.59	0.50	0.27
	**DRM**	**0.69**	**0.47**	**0.74**	**0.80**	**0.58**	**0.72**	**0.59**	**0.46**
**pcaReduce**	**GEM**	0.18	0.16	**0.74**	0.54	0.53	**0.75**	0.54	0.30
	**DRM**	**0.71**	**0.59**	0.73	**0.80**	**0.61**	0.73	**0.59**	**0.44**

Bold values represent larger ARI values.

The DRM also distinguished different cell types more clearly than the GEM in dimension-reduction analysis by t-SNE (Figure 4), as the same cell types are clustered more closely, and different cell types are separated more widely. Notably, the H1 and H9 cells are two very similar types of cells in the Chu-type data which can be distinguished clearly using the DRM but not the GEM.

We used the coefficient of variation (CV) to analyze the feature genes and feature edges. CV is defined as the ratio of the standard deviation to the mean and represents a normalized measure of the dispersion degree of the probability distribution. The top 500 edges and genes with the highest CV were selected. The box plot in Supplementary Figure S2 shows the distribution of the CV of the selected genes and edges on the Chu-type, Yan and Pollen datasets. The CV of edges is significantly higher than that of genes (Wilcoxon rank-sum test, *P*-value <2.2E-16), indicating that edges derived from the DRM contain significantly more information than genes based on the GEM. This may explain why the clustering performance based on the DRM is better than on the GEM.

NDM (network degree matrix) matrix is proposed by Dai [9] by constructing cell-specific network for each cell based on the GEM and then calculating the gene degree of the network to construct the feature matrix. To overcome the overestimation related to indirect effects problem, the CNDM feature was proposed [36]. Moreover, both the NDM and CNDM (feature matrices) performed better than the original GEM in cell clustering. Therefore, we wanted to compare the performance of our DRM to the NDM and CNDM.

The Chu-type, Chu-time and Pollen datasets were used to validate the effectiveness of the DRM in cell cluster analysis. The parameters of all methods were set the same for the DRM, NDM and CNDM. The top 500 features of the DRM, NDM and CNDM were used for cluster analysis unless noted otherwise.

The clustering results based on the DRM are almost superior to those based on the NDM and CNDM in a variety of clustering methods using both the Chu-type data and Pollen data (Figure 5). With the Chu-time data, due to the poor clustering results of the top 500 features of the NDM, we compared clustering results using the top 1000 features of the NDM with those of the top 500 features of the DRM. The third line in Figure 5 shows that the performance of the DRM is still significantly better than that of the NDM and CNDM for all clustering methods and all three evaluation indicators (ARI, NMI and Purity). The second and third lines of Figure 5 show an obvious data bias in the NDM and CNDM. The NDM performs unusually poorly on the Chu-time dataset and similarly to the CNDM on the Pollen dataset. The performance of the DRM, on the other hand, is relatively stable on all three datasets. In addition, we compared the clustering performance for each of the three matrices for all of the features and found that the DRM was almost superior to the NDM and CNDM (Supplementary Figure S3). The construction of the NDM requires a larger memory capacity and longer running time. Using the Chu-time dataset (including 1018 cells) as an example, constructing the DRM takes 192 s, while constructing the NDM requires 38 149 s. The memory occupied by DRM and NDM is 986 MB and more than 2GB, respectively. We used a system with the macOS 10.15.7 with Intel 4-core CPU, a 1 T hard drive and a 8G RAM. In addition, the CNDM has similar memory and time consumption to NDM. In conclusion, our new feature matrix, the DRM, is more suitable for single-cell data analysis in terms of performance and cost.

In addition, we compared the clustering performance of DRM with a method called sciPath [15], which integrated GEM and pathway data. DRM still performed better than sciPaht in cell clustering, although pathway (i.e. gene–gene interaction) information was integrated in sciPath (Supplementary Figure S4).

### The comparison of cell marker edges and cell marker genes

We performed cell marker analysis based on our DRM on the Chu-type dataset [21]. This dataset was obtained from a study of developmental biology and contains seven cell types: H1, H9, HFF, NPC, DEC, EC and TB. Fifty-one cell marker genes have been identified in the Chu-type data by significant difference analysis [21]. We used a similar strategy and identified a similar number of cell marker edges. The top seven significant edges from each cell type were combined into 49 cell marker edges (see Supplementary Table S5) containing 71 genes in total. Moreover, seven genes (*CER1*, *CXCR4*, *EPAS1*, *ERBB4*, *GNG11*, *MAPK10* and *SOX2*) significantly overlapped with the 51 known cell marker genes (*P*-value = 1.67E-06 by hypergeometric test).

The heat maps in Figure 6A and B show the delta rank value of 49 cell marker edges and the expression value of 51 cell marker genes for 1018 cells of seven cell types. Figure 6A shows that different cell types have cell-specific marker edges corresponding to specific delta rank values. It is noteworthy that H1 and H9 cells are very similar and the expression of their cell marker genes is very similar (see Figure 6B). However, for the cell marker edges, we found that the delta rank values of the cell marker edges of H9 cells were significantly different from those of the H1 cells. This may explain why H1 and H9 cells can be distinguished by using the DRM but cannot by using the GEM in Figure 3. In addition, the delta rank of an edge was defined as the difference between the ranks of the two genes connected by the edge, which allows negative and positive values. When the delta rank of cell marker edges for one cell type has a different sign from other cell types, it is easy to separate different cell types.

### Network rewiring on a single cell

To show the power of the DRM for characterizing cell personalized features from a network viewpoint, we chose the Chu-type dataset to analyze the cell-specific subnetworks related to the *NANOG* (or *CDH1*) gene which is involved in human embryo development. The subnetwork of *NANOG* (or *CDH1*) is composed of genes directly connected to *NANOG* (or *CDH1*). Figure 7A shows four cell-specific subnetworks of H1 cells, which clearly characterize cell personalized features. However, the connections between *NANOG* and the *EPAS1*, *FOXD3* and *NR5A1* genes exist in all four subnetworks. Actually, we found that 77.83% of H1 cells include a connection between *NANOG* and *EPAS1*, 21.23% of H1 cells include a connection between *NANOG* and *FOXD3*, and 88.21% of H1 cells include a connection between *NANOG* and *NR5A1*. These connections form the common network pattern for H1 cells related to *NANOG*; however, these connections were rarely found in other types of cells except H9 (see Supplementary Table S6 for specific percentage). *EPAS1* is involved in the development of the embryonic heart and is expressed in endothelial cells in the vascular wall of the umbilical cord [37]. *EPAS1* maintains the stability of catecholamines during early embryonic development to prevent heart failure. *FOXD3* is bound to and activated by transcription from the 6PORE osteopontin enhancer sequence, which is usually expressed early in the blastocyst stage and is thought to be important for blastocyst implantation in the uterine wall [38]. *FOXD3*-overexpressing cells can be maintained for several passages, while downregulation of the transgene leads to further differentiation. Loss of function also leads to differentiation, toward endoderms and mesoderms. A balance of *FOXD3* activity is required to maintain pluripotency [39].

In addition, we analyzed the cell-specific subnetworks of *CDH1* from four TB cells (Figure 7B). These four subnetworks characterize the personalized features of TB cells. However, the connections between *CDH1* and the *RET*, *LEF1*, *MMP7*, *PROC*, *HGF*, *CTNNA3*, *CTNNA2*, *KIFC3* and *ITGB7* genes exist in all four subnetworks. Actually, we found that almost 90% of TB cells include a connection between *CDH1* and the above common genes (see Supplementary Table S7 for specific percentage); however, the proportion of these connections in other types of cells is much lower, revealing that these connections make up the common network pattern for TB cells related to *CDH1*. *RET* homodimerization and phosphorylation can activate multiple signal transduction cascades, induce cell proliferation, and participate in the regulation of cell growth and differentiation. During embryonic development, the *RET* gene plays a key role in the growth and development of the nervous system, kidney, endocrine organs, spermatozoa, etc. [40]. In addition, the *LEF1*, *MMP7*, *PROC*, *HGF*, *KIFC3* and *ITGB7* genes are related to embryonic development [41, 42].

We also performed the network rewiring analysis on the Chu-time developmental biology dataset [21], which contains 758 cells at six time points (0, 12, 24, 36, 72, 96 h) along the differentiation protocol to produce definitive endoderm cells from human embryonic stem cells. We considered the partial cell-specific networks among 12 genes (*BMP4*, *CDH1*, *FOXD3*, *LIN28A*, *NANOG*, *PLAU*, *POU5F1*, *PRDM14*, *SOX2*, *SRC*, *VCL*, *NOG*) involved in human embryo development. Figure 8A shows cell-type-specific subnetworks and illustrates the dynamically changes network topology at different time points. The correlations between the genes were stronger at 12 and 24 h, while the associations weakened considerably after 36 h. The delta rank of edges *NANOG*&*PRDM14*, *POU5F1*&*PRDM14* and *NANOG*&*FOXD3* clearly peaks at 12 h, which means these edges perturb more strongly (i.e. the relative expression orderings of interacted genes change more) and may play important roles from a network point of view at 12 h (Figure 8B). These results imply that the key time point may be around 12 h due to the drastic network rewiring during embryo development.

### Cell pseudo-trajectory analysis

We used the Chu-time dataset [21] with the gold standard stages to perform pseudo-trajectory analysis. It includes 758 human embryonic stem cells in six stages (0, 12, 24, 36, 72, 96 h) [21]. We used Monocle3 to compare the DRM and GEM (Figure 9). In Figure 9B, the brighter the point, the greater its pseudotime values derived from the DRM, which corresponds to the gold standard cell development stage sequence in Figure 9A. However, pseudotime values derived from the GEM do not increase with the continuation of cell differentiation (Figure 9C and D). Instead, the pseudotime values peak at 12 h. The DRM can accurately predict the pseudotime values following the cell developmental stage sequence, but GEM fails (Supplementary Figure S5). This result indicates that the DRM can reconstruct the time series of single cells corresponding to the developmental stages better than GEM.

## Discussion

In this paper, we provided a new feature DRM for single-cell analysis derived from the single-cell GEM using scRNA-seq data. The DRM converts the gene-level information into edge-level information at the single-cell level by integrating an a priori background network, allowing the characterization of a single cell in a biological system. All analyses for dimension-reduction, clustering, cell-marker identification and pseudo-trajectory construction on the GEM also can be performed on DRM from an edge perspective by using any existing scRNA-seq analysis method. Furthermore, we have shown that our new feature DRM is much better than the GEM in almost all performance domains. In cell clustering, the DRM performed better than the GEM in various methods on various datasets as evaluated by ARI, NMI and purity. In addition, the DRM is more effective than the GEM in distinguishing similar types of cells. And experiments on various scRNA-seq datasets validated the effectiveness of the DRM in terms of accuracy and robustness. The excellent performance of the DRM is due to its extracting richer information about biological systems and providing stable characterization for cells (compared with unstable gene expression form) from the perspective of gene–gene interactions. Meanwhile, this stable characterization may remove transcript amplification noise for cells, which is another reason the DRM performed well. Compared with the existing single-cell analysis feature matrix, the NDM, the DRM not only has better performance in cell clustering but also has advantages in time and memory consumption.

Gene–gene interactions are essential for many biological processes such as co-expression, transcriptional regulation, DNA modification and regulation of noncoding RNA. Our new feature DRM creatively provides a method of measuring gene interactions at a single-cell level by changing relative gene expression orderings. Thus, the DRM can be used to find changes in gene interactions among different cell types, just as different expression analyses on the GEM can find differentially expressed genes. This may open up a new method to analyze scRNA-seq data from an interaction perspective.

scRAN-seq data can yield insights into gene–gene interaction networks among cell populations based on the sequencing of a large number of cells. The DRM provides a new method for using scRNA-seq data to construct a cell-specific network for each single cell instead of a group of cells as in traditional network construction methods. Furthermore, our new method does not need more reference samples as background when constructing cell-specific networks. For any given cell transcription profile, a cell-specific network can be constructed. This may lead to a new method for uncovering the heterogeneity of single cells at the network level.

Although the DRM has advantages in many aspects of single-cell analysis, there are still some problems that require further consideration. The construction of the DRM integrates an a priori gene–gene interaction network information. Indeed, an a priori network is important to the construction of the DRM. For species without prior interaction information, our method may not be applicable, so it is important to develop a new strategy to identify useful gene–gene interactions directly from the DRM. In addition, the DRM provides a method for constructing a cell-specific correlation network instead of a causal network. Hence, constructing gene–gene causal relations without an a priori background network for single cells is one of our future topics of research.

**Key Points**

- We construct a new feature matrix called the delta rank matrix (DRM) from scRNA-seq data, which transforms unreliable gene expression values to the stable gene interaction/edge values on a single-cell basis. It is the first time that we have transformed gene-level features into interaction/edge-level for scRNA-seq data analysis based on relative expression orderings.
- Experiments on various scRNA-seq datasets have demonstrated that the DRM performs better than the original gene expression matrix (GEM) in cell clustering, cell identification and pseudo-trajectory reconstruction.
- The DRM achieves true integration of gene expressions and gene interactions and provides a method for measuring gene interactions at the single-cell level. Thus, the DRM can be used to find the changes in gene interactions among different cell types.
- The DRM provides a new method for constructing a cell-specific network for each single cell instead of a group of cells as in traditional network construction methods. This may open up a new way to analyze scRNA-seq data from an interaction perspective.

## Supplementary Material

Supplementary_bbac556

## Availability and implementation

The DRM method introduced in this study has been implemented in R. The source code and used data are available in Supplementary Note S4 and the GitHub websit (https://github.com/xumingmin/DRM).

## Notes

**Yuanyuan Chen** is a postdoc at the State Key Laboratory of Bioelectronics, School of Biological Science and Medical Engineering, Southeast University, Nanjing, China, and an assistant professor at the College of Science, Nanjing Agricultural University, Jiangsu, Nanjing, China.

**Hao Zhang** is a lecturer at the College of Science, Nanjing Agricultural University, Jiangsu, Nanjing, China.

**Xiao Sun** is a professor at the State Key Laboratory of Bioelectronics, School of Biological Science and Medical Engineering, Southeast University, Nanjing, China.
