# Supplementary material for: Improving the performance of single-cell RNA-seq data mining based on relative expression orderings
Source: Brief Bioinform. 2022 Dec 18;24(1):bbac556. doi: 10.1093/bib/bbac556 (PMC9851298; doi:10.1093/bib/bbac556)
Supplement: Supplementary_bbac556 [file supplementary_bbac556.docx]

**Supplementary Materials for**

**“Improving Single-Cell RNA-seq Analysis based on relative expression orderings”**

**Supplementary Figures**


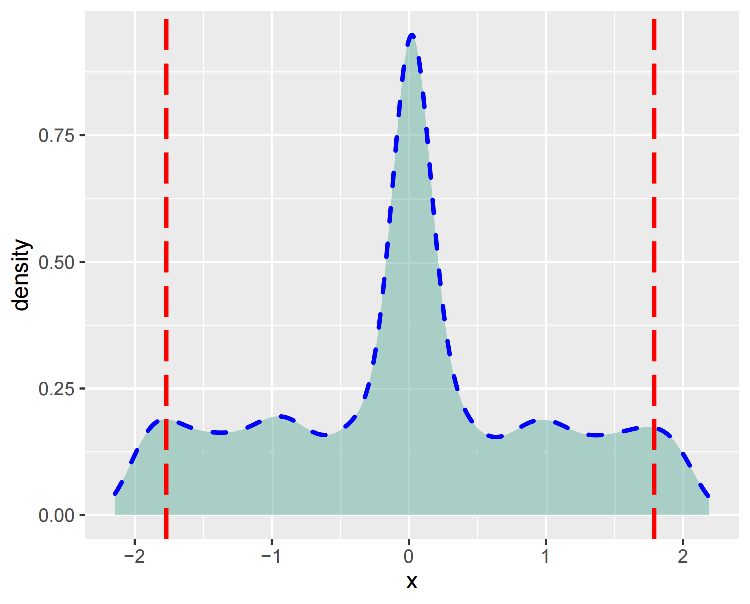


**Figure S1. The empirical NULL distribution of the delta rank value in DRM generated by random shuffling.** The red lines are for the cutoff P-value (0.05) based on left-tailed and right-tailed test respectively.


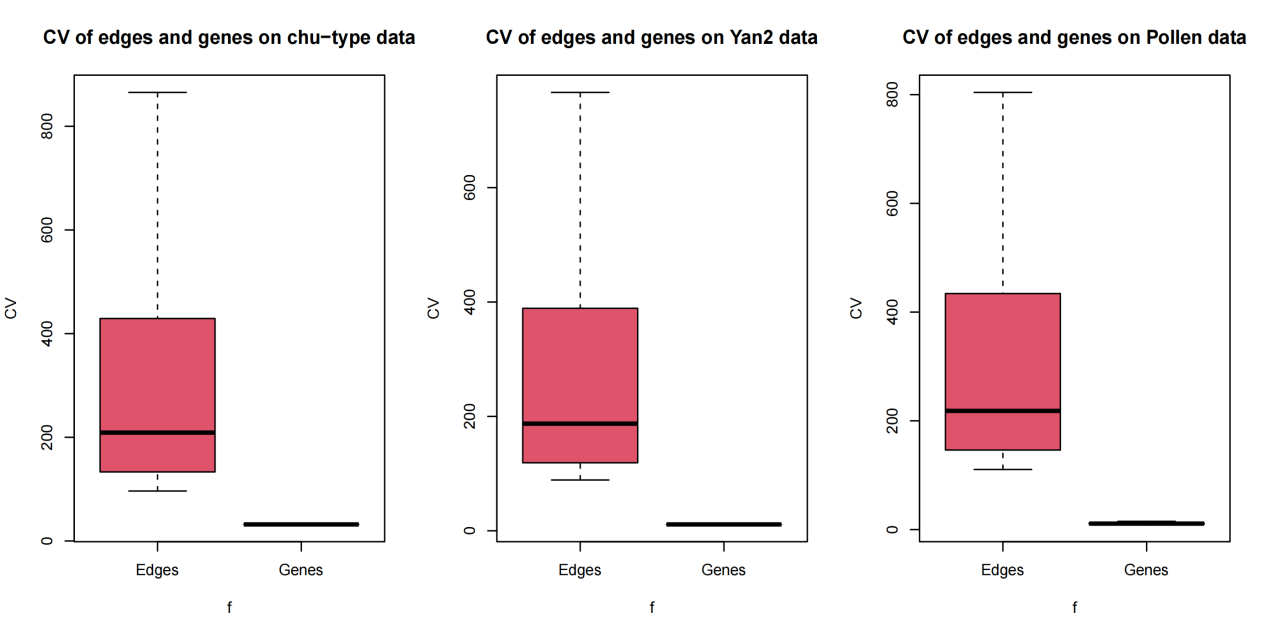


**Figure S2. Coefficient of variantion of edges and genes respect to DRM and GEM respectively on chu-type, Yan2 and Pollen datasets.**

**
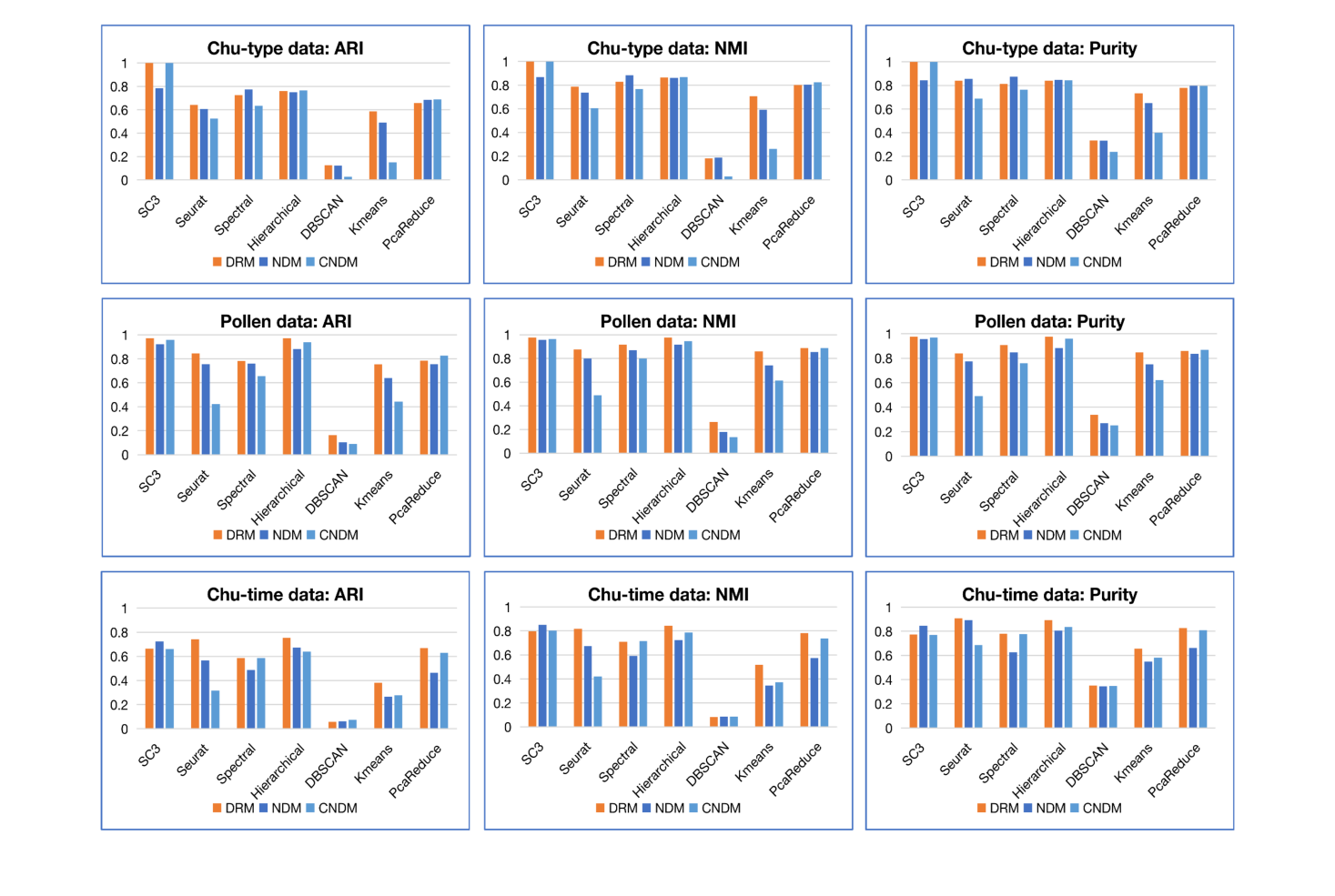
**

**Figure S3. The comparison of the DRM, NDM, and CNDM in cell clustering analysis (using all features), evaluated by ARI, NMI, and Purity on the Chu-type data, Pollen data, and Chu-time datasets.**


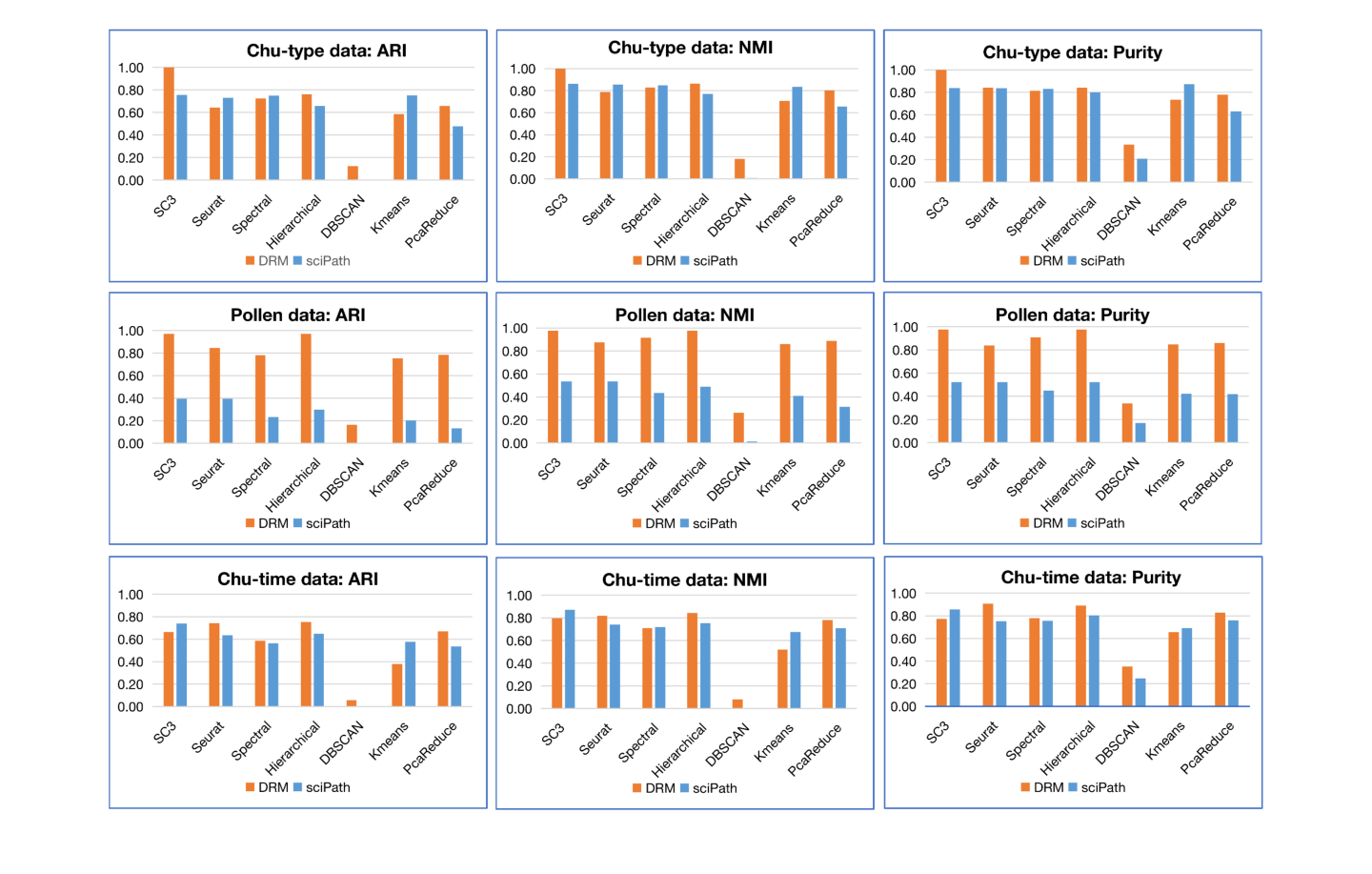


**Figure S4. The comparison of the DRM and sciPath in cell clustering analysis, evaluated by ARI, NMI, and Purity on the Chu-type data, Pollen data, and Chu-time datasets.**The KEGG pathways were integrated in sciPath method.

**
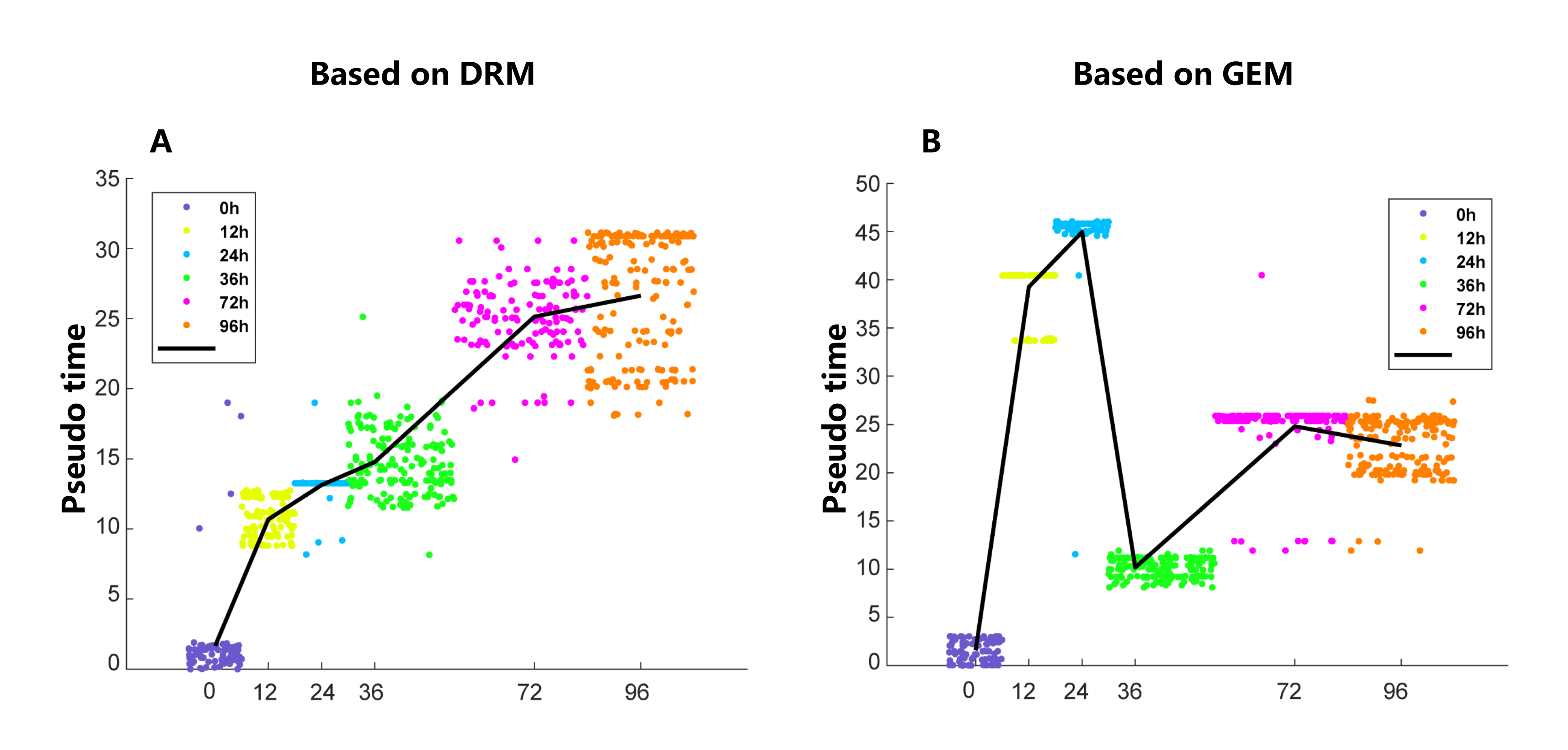
**

**Figure S5. The comparison of DRM and GEM in** **pseudo trajectory analysis.** (A) The pseudotime values of 758 human embryonic stem cells predicted by DRM. (B)The pseudotime values of 758 human embryonic stem cells predicted by GEM. The cells in the later stages will get larger pseudotime values, and the average at every time points is shown as black line. The pseudotime values predicted by DRM increase in accordance with the developmental sequence, but this rule could not be seen in the result predicted by GEM.

**Supplementary Tables**

**Supplementary Table S1. The detail of scRNA-seq datasets from normal human liver tissues.** Four scRNA-seq datasets are used for validating the stability of relative expression ordering of gene pairs.

| **Dataset** | **Number of cell types** | **Number of cells/**  **Hepatocyte cells** | **platform** | **Experimental protocol** | **Data Sources** |
| --- | --- | --- | --- | --- | --- |
| Aizarani [15] | 8 | 10197/2977 | [GPL16791](https://www.ncbi.nlm.nih.gov/geo/query/acc.cgi?acc=GPL16791) Illumina HiSeq 2500 | mCel-Seq2 | GSE124395 |
| MacParland [16] | 9 | 7826/3220 | [GPL16791](https://www.ncbi.nlm.nih.gov/geo/query/acc.cgi?acc=GPL16791) Illumina HiSeq 2500 | 10X Genomics (Hi-seq) | GSE115469 |
| Li [17] | 8 | 19630/75 | [GPL24676](https://www.ncbi.nlm.nih.gov/geo/query/acc.cgi?acc=GPL24676) Illumina NovaSeq 6000 | 10x Genomics | GSE149614 |
| Ramachandran [18] | 9 | 32086/36 | [GPL20301](https://www.ncbi.nlm.nih.gov/geo/query/acc.cgi?acc=GPL20301) Illumina HiSeq 4000 | Drop-seq | GSE136103 |

**Supplementary Table S2. The detail of Single-cell RNA-seq datasets.** Eight single-cell RNA-seq datasets are used for validating the new feature matrix DRM in this paper.

| **Dataset** | **Number of cell types** | **Number of cells** | **Normalization method** | **Data Sources** |
| --- | --- | --- | --- | --- |
| Chu-type[1] | 7 | 1018 | FPKM | GSE75748 |
| Chu-time[1] | 6 | 758 | FPKM | GSE75748 |
| Yan[2] | 6 | 90 | RPKM | GSE36552 |
| Yan2[2] | 8 | 124 | RPKM | GSE36552 |
| Camp[3] | 7 | 770 | FPKM | GSE81252 |
| Pollen[5] | 11 | 249 | TPM | NCBI Sequence Read Archive：SRP041736 |
| Darmanis[6] | 8 | 420 | TPM | GSE67835 |
| Calzetti[7] | 12 | 44 | FPKM | GSE175880 |

**Supplementary Table S3. The comparison of GEM and DRM in clustering analysis, evaluated by NMI.**

|  |  | **Chu-type** | **Chu-time** | **Yan1** | **Yan2** | **Camp2** | **Pollen** | **Darmanis** | **GSE175880** |
| --- | --- | --- | --- | --- | --- | --- | --- | --- | --- |
| **SC3** | **GEM** | 0.86 | **0.86** | 0.79 | 0.74 | **0.82** | **0.94** | **0.85** | 0.70 |
|  | **DRM** | **0.99** | 0.83 | **0.83** | **0.82** | 0.80 | 0.92 | 0.80 | **0.73** |
| **Seurat** | **GEM** | 0.70 | 0.63 | 0.66 | 0.67 | **0.77** | 0.82 | 0.79 | 0.25 |
|  | **DRM** | **0.87** | **0.78** | **0.66** | **0.74** | 0.76 | **0.83** | **0.79** | **0.25** |
| **spectral** | **GEM** | 0.40 | 0.35 | 0.92 | 0.85 | **0.74** | 0.79 | 0.63 | 0.71 |
|  | **DRM** | **0.86** | **0.64** | **0.92** | **0.92** | 0.73 | **0.87** | **0.64** | **0.76** |
| **hierarchical** | **GEM** | 0.46 | 0.35 | **0.89** | 0.76 | 0.79 | 0.91 | **0.69** | 0.62 |
|  | **DRM** | **0.86** | **0.80** | 0.78 | **0.84** | **0.81** | **0.92** | 0.73 | **0.78** |
| **DBSCAN** | **GEM** | 0.10 | 0.02 | 0.71 | 0.26 | 0.23 | 0.14 | 0.06 | **0.22** |
|  | **DRM** | **0.77** | **0.37** | **0.72** | **0.75** | **0.23** | **0.80** | **0.03** | 0 |
| **kmeans** | **GEM** | 0.23 | 0.26 | 0.78 | 0.66 | 0.64 | 0.69 | 0.53 | 0.67 |
|  | **DRM** | **0.79** | **0.62** | **0.79** | **0.83** | **0.65** | **0.84** | **0.65** | **0.77** |
| **pcaReduce** | **GEM** | 0.33 | 0.28 | **0.82** | 0.69 | 0.66 | 0.82 | 0.64 | 0.69 |
|  | **DRM** | **0.85** | **0.71** | 0.80 | **0.84** | **0.70** | **0.86** | **0.69** | **0.75** |

**Supplementary Table S4. The comparison of GEM and DRM in clustering analysis, evaluated by purity.**

|  |  | **Chu-type** | **Chu-time** | **Yan1** | **Yan2** | **Camp2** | **Pollen** | **Darmanis** | **GSE175880** |
| --- | --- | --- | --- | --- | --- | --- | --- | --- | --- |
| **SC3** | **GEM** | 0.84 | **0.85** | 0.84 | 0.82 | **0.80** | **0.95** | **0.91** | 0.61 |
|  | **DRM** | **0.99** | 0.82 | **0.87** | **0.89** | 0.77 | 0.91 | 0.86 | **0.70** |
| **Seurat** | **GEM** | 0.81 | **0.82** | 0.68 | 0.72 | 0.76 | 0.77 | **0.89** | **0.20** |
|  | **DRM** | **0.83** | 0.80 | **0.68** | **0.77** | **0.77** | **0.78** | 0.87 | 0.18 |
| **spectral** | **GEM** | 0.53 | 0.45 | 0.94 | 0.93 | 0.75 | 0.81 | 0.78 | 0.57 |
|  | **DRM** | **0.84** | **0.70** | **0.94** | **0.96** | **0.75** | **0.85** | **0.80** | **0.66** |
| **hierarchical** | **GEM** | 0.53 | 0.46 | **0.91** | 0.83 | 0.77 | **0.93** | 0.83 | 0.55 |
|  | **DRM** | **0.84** | **0.80** | 0.84 | **0.89** | **0.77** | 0.92 | **0.88** | **0.68** |
| **DBSCAN** | **GEM** | 0.29 | 0.29 | **0.78** | 0.40 | 0.36 | 0.26 | **0.36** | **0.25** |
|  | **DRM** | **0.77** | **0.43** | 0.77 | **0.80** | **0.36** | **0.76** | 0.34 | 0.11 |
| **kmeans** | **GEM** | 0.40 | 0.38 | 0.80 | 0.75 | 0.67 | 0.71 | 0.74 | 0.56 |
|  | **DRM** | **0.79** | **0.68** | **0.85** | **0.88** | **0.69** | **0.80** | **0.82** | **0.69** |
| **pcaReduce** | **GEM** | 0.47 | 0.43 | **0.86** | 0.76 | 0.68 | 0.82 | 0.79 | 0.59 |
|  | **DRM** | **0.83** | **0.77** | 0.85 | **0.91** | **0.71** | **0.82** | **0.84** | **0.66** |

**Supplementary Table S5. The 49 identified cell marker edges for Chu-type data.** Here the edges are denoted by genes pairs. Gene 1 and Gene 2 in a row are tow genes connected by an edge.

| **Gene1** | **Gene2** |  | **Gene1** | **Gene2** |  | **Gene1** | **Gene2** |
| --- | --- | --- | --- | --- | --- | --- | --- |
| CXCL12 | ERBB4 |  | AURKB | VIM |  | CAV1 | FZD5 |
| DCN | ERBB4 |  | POLR3G | POLR3GL |  | FOXO1 | MAPK10 |
| CXCL12 | CXCR4 |  | POLR3G | SNAPC3 |  | CD44 | COL2A1 |
| ERBB2 | ERBB4 |  | CAMK2D | GRIA4 |  | FOS | MAPK10 |
| ERBB4 | RASAL2 |  | RPS4Y1 | SEC11C |  | GNG11 | MAPK10 |
| CYP26A1 | CYP2S1 |  | RPS4Y1 | UPF3B |  | DUSP5 | MAPK10 |
| CER1 | WNT5B |  | EIF3B | RPS4Y1 |  | DUSP6 | MAPK10 |
| ACTC1 | CDH5 |  | EIF4A3 | RPS4Y1 |  | COL2A1 | PLOD2 |
| CDH5 | CTNNAL1 |  | ETF1 | RPS4Y1 |  | CCND1 | THRB |
| RASGRP3 | RRAS2 |  | CASC3 | RPS4Y1 |  | EGLN3 | EPAS1 |
| LPL | SDC1 |  | GSPT2 | RPS4Y1 |  | FZD7 | PLCB1 |
| CDH13 | CTNNAL1 |  | CCND2 | CDKN1A |  | GUCY1A3 | POLR3G |
| EFNA1 | INSR |  | FGF12 | PDGFRA |  | EPAS1 | SOX2 |
| LPL | MED21 |  | CA2 | COL6A3 |  | COL3A1 | SDC1 |
| ATP2B4 | TRDN |  | EFNA5 | PDGFRA |  | ANXA1 | GNG11 |
| NFIC | POLR3G |  | B3GNT7 | LUM |  |  |  |
| PHC1 | PHC2 |  | CA2 | COL3A1 |  |  |  |

**Supplementary Table S6. The percentage of H1 cells with a connection between NANOG and the common genes EPAS1, FOXD3 and NR5A1.**

| **Connections** | | | |
| --- | --- | --- | --- |
| **Celltype** | NANOG  &  EPAS1 | NANOG  &  FOXD3 | NANOG  &  NR5A1 |
| **DE** | 0.0942 | 0.0942 | 0.1014 |
| **EC** | 0 | 0.0095 | 0.0190 |
| **H1** | **0.7830** | **0.2123** | **0.8821** |
| **H9** | 0.7469 | 0.3025 | 0.8457 |
| **HF** | 0 | 0 | 0 |
| **NP** | 0.0173 | 0.0173 | 0.0289 |
| **TB** | 0 | 0 | 0 |

**Supplementary Table S7. The** **percentage of TB cells with a connection between CDH1 and the common genes** (including RET, LEF1, MMP7, PROC, HGF, CTNNA3, CTNNA2, KIFC3 and ITGB7).

|  | **Connections** | | | | | | | | | |
| --- | --- | --- | --- | --- | --- | --- | --- | --- | --- | --- |
| **Celltype** | CDH1  &  RET | CDH1  &  LEF1 | CDH1  &  HGF | CDH1  &  MMP7 | CDH1  &  PROC | CDH1  &  CTNNA2 | CDH1  &  CTNNA3 | CDH1&  KIFC3 | CDH1  &  ITGB7 | CDH1&  RET |
| **DE** | 0.0870 | 0.0870 | 0.0870 | 0.0870 | 0.0870 | 0.0725 | 0.0798 | 0.0363 | 0.0798 | 0.0870 |
| **EC** | 0 | 0 | 0 | 0 | 0 | 0 | 0 | 0 | 0 | 0 |
| **H1** | 0.6792 | 0.7123 | 0.7453 | 0.7547 | 0.7453 | 0.4575 | 0.6321 | 0.6887 | 0.7406 | 0.6792 |
| **H9** | 0.4877 | 0.5864 | 0.6296 | 0.6296 | 0.6296 | 0.3642 | 0.5802 | 0.5864 | 0.6111 | 0.4877 |
| **HF** | 0 | 0 | 0 | 0 | 0 | 0 | 0 | 0 | 0 | 0 |
| **NP** | 0 | 0 | 0 | 0 | 0 | 0 | 0 | 0 | 0 | 0 |
| **TB** | **0.9565** | **0.5362** | **0.9565** | **0.9565** | **0.9420** | **0.9275** | **0.8261** | **0.7971** | **0.9565** | **0.9565** |

**Supplementary Notes**

**Supplementary Note S1. The detail of Single-cell RNA-seq datasets.** Eight single-cell RNA-seq datasets are used for validating the new feature matrix DRM in this paper.

The Chu-type dataset [20] includes 1018 cells and 7 cell types. It contains the cells from human embryonic stem cell-derived lineage-specific progenitors. Cell types including H1 embryonic stem cells (H1), H9 embryonic stem cells (H9), human foreskin fibroblasts (HFF), neuronal progenitor cells (NPC), definitive endoderm cells (DEC), endothelial cells (EC) and trophoblast-like cells (TB) were identified by FACS with their respective gene markers.

The Chu-time dataset [20] includes 758 cells and 6 cell types. This dataset contains cells from 6 time points along the differentiation protocol to produce definitive endoderm cells from human embryonic stem cells. A total of 758 cells were captured and profiled by scRNA-seq at 0, 12, 24, 36, 72 and 96 h of differentiation.

The Yan dataset [21] includes 124 cells and 8 cell types from human preimplantation embryos and human embryonic stem cells (hESCs) at different passages. The cell types include the cells of oocyte cells, zygote cells, 2-cell, 4-cell, 8-cell, morula cells, late blastocyst cells, and hESC cells.

A subset of the Yan2 dataset [21] contains 90 cells and 7 cell types from human preimplantation embryos, excluding the hESC cells.

The Camp dataset [22] includes 770 cells and 7 cell types. This dataset contains, cells from multiple time points during a hepatocyte-like lineage progression from pluripotency in 2-D culture and 3-D liver bud (LB) organoids that self-organized after reconstituting hepatic, stromal, and endothelial interactions. The cell types include definitive endoderm cells (DE), mature hepatocyte-like cells (MH), immature hepatoblast-like cells (IH), hepatic endoderm cells (HE), induced pluripotent stem cells (iPS), endothelial cells (EC), and mesenchymal cells (MC).

The Pollen dataset [23] includes 249 cells and 11 cell types. This dataset includes skin cells, pluripotent stem cells, blood cells, and neural cells, and was designed to test the utility of low-coverage single-cell RNA-seq in identifying distinct cell populations.

The Darmanis dataset [24] includes 420 cells and 8 cell types. This dataset contains the cells of human cortical tissue cells from eight adults and four embryonic samples. The cell types including OPCs, oligodendrocytes, astrocytes, microglia, neurons, endothelial cells, replicating neuronal progenitors and quiescent newly born neurons were identified through unbiased clustering and validated by using some gene markers derived from the mouse brain.

The Calzetti dataset [25] includes 44 cells and 12 cell types, including neutrophil myeloblasts (NMs), promyelocytes (PMs), myelocytes (MYs), metamyelocytes (MMs), band cells (BCs), and segmented neutrophils (SNs) from bone marrows as well as peripheral blood neutrophils, isolated by cell sorting by using a Becton Dickinson FACS Aria Fusion (BD Biosciences).

**Supplementary Note S2. The default parameters used in the cell clustering methods.**

1. **SC3**

Available packages: *R package ‘SC3’ (v1.10.1), “hclust” function*

Number of clusters: *Same as the number of categories in the original dataset*

Other parameters:*‘gene_filter’ = False, and other parameters remain Default*

1. **Seurat**

Available packages: *R package ‘Seurat’ (v3.0.0.9110), “FindClusters” function*

Number of clusters: *Same as the number of categories in the original dataset*

Other parameters: *Default*

1. **Spectral**

Available packages: *R package ‘SNFtool’ (v2.3.0), “spectralClustering” function*

Number of clusters: *Same as the number of categories in the original dataset*

Other parameters: *Default*

1. **Hierarchical**

Distance between two objects: *Euclidean distance*

Algorithm for computing distance between clusters: *Ward’s linkage* (Inner squared distance,

minimum variance algorithm)

Available packages: *R package ‘fastcluster’ (v1.1.25), “hclust” function*

Number of clusters: *Same as the number of categories in the original dataset*

1. **DBSCAN**

Available packages: *R package ‘dbscan’ (v1.1-3), “dbscan” function*

Number of clusters: *Same as the number of categories in the original dataset*

Other parameters: *‘eps’ = 5%, ‘k’ = 5, others Default*

1. **Kmeans**

Distance between two objects: *Squared Euclidean distance*

Available packages: *R package ‘stats’ (v3.5.2), “kmeans” function*

Maximum number of iterations: *1000*

Number of times to repeat clustering using new initial cluster centroid positions: *100*

Number of clusters: *Same as the number of categories in the original dataset*

1. **pcaReduce**

Available packages: *R package ‘pcaReduce’ (v1.0), “dbscan” function*

Number of clusters: *Same as the number of categories in the original dataset*

Other parameters: *‘nbt’ = 5, ‘q’ = #cluster-1 and ‘method’ = ‘s’, others Default*

**Supplementary Note S3. The details of the cell clustering methods used in this paper.**

SC3 [26]. A single-cell unsupervised clustering method, integrates many different clustering solutions through a consensus approach, thereby increasing its accuracy and robustness against noise. The R packages ‘SC3’ (v1.10.1) used in this paper is available at http://bio conductor.org/packages/sc3. The ‘gene_filter’ parameter is the bool value that controls whether gene filtering is performed in data preprocessing (‘gene_filter’ = False) and other processes and parameters remain default.

Seurat [27]. The modularity optimization of cell–cell network which is constructed by SNN after feature selection (high variance gene selection) and feature extraction (principal component analysis). The R packages ‘Seurat’ (v.3) used in this paper is available at https://cran.r-project.org/package=Seurat. The ‘FindClusters’ function was used to cluster cells and all processes and parameters remain default.

Hierarchical clustering [28]. Assuming that each sample is a separate cluster class, and then the cluster with high similarity is found and merged in each iteration of the algorithm. The process is repeated until the preset cluster number K is reached or there is only one cluster. The ‘hclust’ function with ‘ward.D’ agglomeration method and Euclidean distance in ‘fastcluster’ (v1.1.25) R package used in this paper is available at https://cran.r-project.org/web/packages/fastcluster/index.html.

Spectral clustering [29]. Clustering idea is using the similarity matrix (Laplace matrix) to carry on the clustering of the eigenvectors obtained after the eigendecomposition. The ‘spectralClustering’ function in R package ‘SNFtool’ (v2.3.0) was used at <https://cran.r-project.org/web/packages/SNFtool/index.html.>

DBSCAN [30]. As a representative clustering algorithm based on density, it defines the cluster as the maximum set of points connected by density. It can divide the region with sufficient density into clusters, and arbitrary shape clusters in the spatial database of noise can be found. The ‘dbscan’ function in R package ‘dbscan’ (v1.1-3) was used at https://cran.r-project.org/web/packages/dbscan/index.html. The parameter ‘eps’ in ‘dbscan’ function indicates the radius of neighbor selection, obtained by extracting the 5–95% (with 5% interval) quantile knn distance(‘kNNdist’ function with k = 5) between cells.

K-means [31]. Given the number of clusters k, constructing k points as the cluster centers and calculating the distance of each same to each the cluster center, updating the attribution of samples and the cluster center until both are stable. The ‘kmeans’ function in R package ‘stats’ (v3.5.2) was used.

pcaReduce [32]. An agglomerative algorithm to differentiate cell states by generating a cell state hierarchy where each cluster branch is associated with a principal component of variation. The R package ‘pcaReduce’ (v1.0) used in this paper is available at https://github.com/JustinaZ/pcaReduce. The parameters ‘nbt’, ‘q’ and ‘method’ indicate runs, number of cluster and merging strategy, respectively. (‘nbt’ = 5, ‘q’ = #cluster-1 and ‘method’ = ‘s’ in our experiment).

**Supplementary Note S4. Source code (R).**

################################################################################

#' @description: convert Gene Expression Matrix to Rank Matrix denoted by R

#' with element r_is, which represents the rank of gene g_i in cell s

#' @param: X, Expression matrix data of samples (comprises of m genes and n cells)

#' @return: Rank Matrix (m*n)

################################################################################

rank.matrix <- function(x){

rankmatrix = apply(x, 2, rank)

colnames(rankmatrix) = colnames(x)

row.names(rankmatrix) = row.names(x)

return(rankmatrix)

}

###############################################################################

#' @description: Caculate the Delta Rank Matrix for all cells based on a background network and #' the rank matrix

#' @param: net, the backgound network with l edges (two column genes connected in the

#' background network)

#' @param: rank.data, Rank Matrix (m*n dimension, m genes and n cells)

#' @return: the Delta Rank Matrix, l*n dimension, l edges and n cells

###############################################################################

delta.rank <- function(rank.data, net){

deltarank <- rank.data[net[,1],]-rank.data[net[,2],]

rownames <- paste(net[,1],net[,2],sep="|")

row.names(deltarank) <- rownames

colnames(deltarank) <- colnames(rank.data)

return(deltarank)

}

################################################################################

#' @description: construction of cell-specific network

#' @param: deltarank, normalized Delta Rank Matrix

#' @param: alpha: Significant level (eg. 0.001, 0.01, 0.05 ...), larger alpha leads to more edges, Default = 0.05

#' @return: CSN, cell-specific network, 0-1 matrix (if an edge exists in a cell, the value is 1; ortherwise, 0.

#################################################################################

cutoff <- c()

for (i in 1:1000){

cutoff <- rbind(cutoff, quantile(deltarank[sample(1:nrow(deltarank)*ncol(deltarank), 10000)], probs = c(alpha, 1-alpha)))

}

cutoff <- apply(cutoff, 2, mean)

index <- c(which(deltarank > cutoff[2]), which(deltarank < cutoff[1]))

CSN <- matrix(data=NA, nrow = nrow(deltarank), ncol = ncol(deltarank), byrow = FALSE)

CSN[index] <- 1

CSN[-index] <- 0

rownames(CSN) <- rownames(deltarank)

colnames(CSN) <- colnames(deltarank)
